# Albumin alleviated esketamine-induced neuronal apoptosis of rat retina through downregulation of Zn^2+^-dependent matrix metalloproteinase 9 during the early development

**DOI:** 10.1186/s12868-022-00753-5

**Published:** 2022-11-16

**Authors:** Kan Zhang, Ruijing Ma, Luping Feng, Peiwen Liu, Shuang Cai, Chaoyang Tong, Jijian Zheng

**Affiliations:** 1 Department of Anesthesiology & Laboratory of Pediatric Clinical Pharmacology, Shanghai Children’s Medical Center, Shanghai Jiao Tong University School of Medicine, Shanghai, 200127 China; 2 Center for Brain Science, Shanghai Children’s Medical Center, Shanghai Jiao Tong University School of Medicine, Shanghai, 200127 China; 3 Department of Anesthesiology, Shanghai First Maternity and Infant Hospital, School of Medicine, Tongji University, Shanghai, 200127 China

**Keywords:** Albumin, Apoptosis, Esketamine, Matrix metalloproteinase 9, Zn^2+^

## Abstract

**Aims:**

Esketamine upregulates Zn^2+^-dependent matrix metalloproteinase 9 (MMP9) and increases the neuronal apoptosis in retinal ganglion cell layer during the early development. We aimed to test whether albumin can alleviate esketamine-induced apoptosis through downregulating Zn^2+^-dependent MMP9.

**Methods:**

We investigate the role of Zn^2+^ in esketamine-induced neuronal apoptosis by immunofluorescence. MMP9 protein expression and enzyme activity were investigated by zymography in situ*.*, western blot and immunofluorescence. Whole-mount retinas from P7 Sprague-Dawley rats were used.

**Results:**

We demonstrated that esketamine exposure increased Zn^2+^ in the retinal GCL during the early development. Zn^2+^-dependent MMP9 expression and enzyme activity up-regulated, which eventually aggravated apoptosis. Albumin effectively down-regulated MMP9 expression and activity via binding of free zinc, ultimately protected neurons from apoptosis. Meanwhile albumin treatment promoted activated microglia into multi-nucleated macrophagocytes and decreased the inflammation.

**Conclusion:**

Albumin alleviates esketamine-induced neuronal apoptosis through decreasing Zn^2+^ accumulation in GCL and downregulating Zn^2+^-dependent MMP9.

**Supplementary Information:**

The online version contains supplementary material available at 10.1186/s12868-022-00753-5.

## Introduction

Since the U.S. Food and Drug Administration (FDA) first released the warning that repeated or lengthy use of general anesthetic and sedation drugs during surgeries or procedures in children younger than 3 years or in pregnant women during their third trimester may affect the development of children’s brains in 2016, many clinical trials and animal studies have been conducted to investigate this issue. Although some clinical trials found that short-term exposure to general anesthesia in children less than 3 years might not affect cognitive changes [1–3], lots of animal studies (*e.g.,* rodents and non-human primates) have found that long-time or repeated general anesthesia during early development stage can aggravate neuronal apoptosis and hypomyelination [4–7]. Furthermore, recent clinical trials demonstrate that children exposed to general anesthesia in the early life are associated with lower motor and social communication performance instead of lower global Intelligence quotient (IQ) [8, 9], indicating that general anesthesia exposure can cause some specific neurotoxicity during the early life.

Although the precise mechanism of general anesthesia-induced neurotoxicity during the early development is unclear, neuronal apoptosis is one of the most basic characteristic induced by general anesthetics through impairing neurotrophy signaling, blockade of synchronized network activity, neuroinflammation and remodeling of the extracellular matrix (ECM) [4, 10–15], etc. As one of the most commonly used general anesthetics in children before, our previous study demonstrated that ketamine aggravated neuronal apoptosis by MMP9 upregulation and ECM degradation during the early development of rat retina. Furthermore, chelator TPEN decreased free Zn^2+^, inhibited upregulation of MMP9 and ECM degradation, and protected neuron [15]. However, TPEN were non-clinical agents and not allowed for treatment, especially in neonates and infants.

Albumin infusion is a commonly used therapy for fluid resuscitation and for patients with severe hypoalbuminemia, which has a potent binding capacity to metabolites and metals. Generally, approximately 75% of the total 15–20 mM Zn^2+^ in plasma is bound to serum albumin, constituting > 99% of the mobile Zn^2+^ pool, which is considered a key regulator in the homeostasis of zinc. However, whether albumin can inhibit the MMP9 through reducing free Zn^2+^ and ameliorate general anesthesia induced neuronal apoptosis remain elusive.

In current study, we aimed to explore the effects of esketamine, an active enantiomer of ketamine in terms of NMDA receptor antagonism and more potent than racemic ketamine, on neuronal apoptosis during the early development of rat retina and to investigate whether albumin can ameliorate esketamine-induced neuronal apoptosis through inhibition of Zn^2+^ accumulation and ECM degradation caused by downregulation of Zn^2+^-dependent MMP9.

## Methods

### Animals

All experiments were completed according to the Animal Care Committee of Shanghai Children’s Medical Center, Shanghai Jiao Tong University School of Medicine. All methods are reported in accordance with ARRIVE guidelines (https://arriveguidelines.org) for the reporting of animal experiments. Sprague Dawley rat pups were from the Experimental Animal Center of Shanghai Children’s Medical Center. Male and female rats were included in the current study. Rat pups were housed with their mothers and under a 12-h light-dark cycle.

### Drugs and chemicals

The following drugs were used: esketamine, the pure dextrorotatory enantiomer of esketamine (150 μM, Hengrui Pharmaceutical Co.). Bovine serum albumin (BSA, absin), ZnCl_2_ (100 μM, Sangon Biotech), Tris (2-butoxyethyl) phosphate (TBEP, 10 μM, Sigma-Aldrich), N, N, N’, N’-tetrakis (2-pyridylmethyl) ethylenediamine (TPEN, 100 μM, Med Chem Express). Artificial cerebrospinal fluid (ACSF) was used to dissolve those drugs.

### Tissue dissection

Retinal tissues were prepared as previously described [14]. In short, rats on postnatal day (P) 7 were decapitated instantaneously in accordance with the Guidelines for the Care and Use of Laboratory Animals Protocols from the US National Institutes of Health. The eyeballs were removed rapidly using fine scissors and dissected in an ice-cold bath of ACSF. The composition of ACSF was as follows (in mmol/L): 119 NaCl, 26.2 NaHCO_3_, 2.5 KCl, 1.3 MgCl_2_, 11 D-glucose, 1.0 KH_2_PO_4_, and 2.5 CaCl_2_, equilibrated with 95% O_2_ and 5% CO_2_, pH 7.4. To facilitate full exposure of the retina to drugs, an incision spanning approximately 1/5th of the circumference of the eyeball was made between the edges of the cornea and sclera. After 1 h of recovery in ACSF bubbled with a mixture of 95% O_2_ and 5% CO_2_ gas at 37 ℃, the eyeballs were incubated with esketamine and/or albumin etc. in ACSF for 5 h.

### Immunofluorescence

The whole-mount retinas dissected were fixed in 4% paraformaldehyde (PFA) at 4 ℃ overnight and then dehydrated with 30% sucrose for 24 h. Retinas were embedded in OCT compound and cut into 20-μm-thick slices using Leica CM1950 cryostat. Frozen sections were incubated in permeabilizing buffer (0.3% Triton X-100 in PBS) for 15 min and then in blocking buffer (Beyotime) for 2 h at room temperature (RT). Sections were incubated overnight at 4 °C with primary antibodies as follows: rabbit antibody to MMP9 (1:400, Abcam ab38898), mouse antibody to NeuN (1:400, Merk MAB377), cleaved caspase 3 (1:400, Cell Signaling 9661S). Sections were incubated 2 h at RT with secondary antibodies as follows: Donkey anti-Rabbit Alexa Fluor 594 (1:500, Abcam ab150064) and Donkey anti-mouse Alexa Fluor 488 (1:500, Abcam ab150109). Sections were incubated with DAPI (1:1000, Cell Signaling 4083 S) for 15 min. Fluorescent images were visualized using a confocal microscope (Leica TCS SP8). Images for analysis of fluorescence intensity were under the same exposure. The cells of interest were counted according to the DAPI channel threshold image. Image analysis was performed by ImageJ (US National Institutes of Health).

### 3D reconstruction

For 3D reconstruction of confocal images from rat retina, the sections were stained with anti-NeuN (1:400, Abcam ab13970) and anti-MMP9 (1:400, Synaptic Systems 147,011). The confocal Z stack images were captured every 1 μm consecutively on a Leica SP8 confocal microscope (Leica, Tokyo, Japan) with oil objective (HC PL APO CS2 × 63/1.40). The Z stack images were simulated to 3D movies using LAS X software (Leica Microsystems).

### in situ Gelatin substrate zymography

Fluorescent in situ gelatin substrate zymography (Genmed, GMS80062.1) was used to measure the proteolytic activity of MMP9 [15]. Unfixed and OCT-embedded retinas were cut into 10 μm-thick slices. Reagent B was warmed to room temperature in the dark, and Reagent A was heated in hot water. Following this, 400 mL Reagent A was placed in a 1.5 mL centrifuge tube and incubated for 10 min at 37 °C. Then, 50 mL reagent B preheated at 37 °C was added to reagent A. Subsequently, 40 mL of this mixed liquid was immediately added to frozen sample. The samples were then covered with coverslips and incubated in a 4 °C refrigerator for 10 min until the colloid coagulated. Finally, the tissue samples were incubated in a 37 °C incubator for 1 h. The fluorescence intensity of the samples was captured under the same exposure via confocal microscopy.

### Zn^2+^ fluorescence in retina and quantitation of intensity

Zinpyr-1 is a selective and membrane-permeable fluorescent probe for Zn^2+^. The K_d_ for Zn^2+^ is 0.7 ± 0.1 nM and has no detectable interaction with Ca^2+^ [16, 17]. Unfixed and OCT-embedded retinas were cut into 16 μm-thick slices. A 1 mM stock solution of Zinpyr-1 (Abcam, ab145349; excitation/emission: 490/530 nm) was prepared in DMSO; a 17 μM working solution was then made using 0.9% saline. Retina sections on slides were immediately covered in working solution for three minutes in darkness and after were tilted to remove the Zinpyr-1 solution. Fluorescent images were captured and analyzed by methods mentioned above.

### Western blotting

The protein concentration of retinal extracts was tested using a BCA kit (Beyotime Biotechnology), and 40 μg of proteins in 10% SDS-PAGE was electrophoresed. The separated proteins were then transferred to nitrocellulose membranes (Millipore). Bovine serum albumin in Tris-buffer saline was used to block nonspecific binding. The membranes were incubated with anti-MMP9 at 4 °C overnight. β-actin was used as a loading control. The membranes were then incubated with a horseradish peroxidase-streptavidin-conjugated secondary antibody for 2 h at room temperature. Antibody detection was performed via enhanced chemiluminescence (Thermo Fisher Scientific), and the intensity of the bands was quantified by densitometric analysis using ImageJ (US National Institutes of Health) [15].

### Statistical analysis

All experiments were performed with at least three biological replicates independently as indicated in the figure legends. All statistical tests were performed in GraphPad Prism 8 or IBM SPSS Statistics 23 (SPSS Inc, IBM Corporation). Data are shown as mean ± SD. For each set of data to be compared, we first examined whether the data were distributed normally. If they were normal distribution, we used unpaired Student’s two-tailed t tests or ANOVA with Bonferroni multiple comparisons post hoc were used as appropriate. If the data were not normal distribution, we used two-tailed Mann–Whitney test. For all biochemistry, n values represent the number of retinas [18]. Investigators were blind to the groups or samples during the experiments. Statistical significance was set at */† P < 0.05, **/†† P < 0.01, ***/††† P < 0.001.

## Results

### Esketamine induces apoptosis and MMP9 upregulation in retinal GCL during the early development

**Fig. 1:**
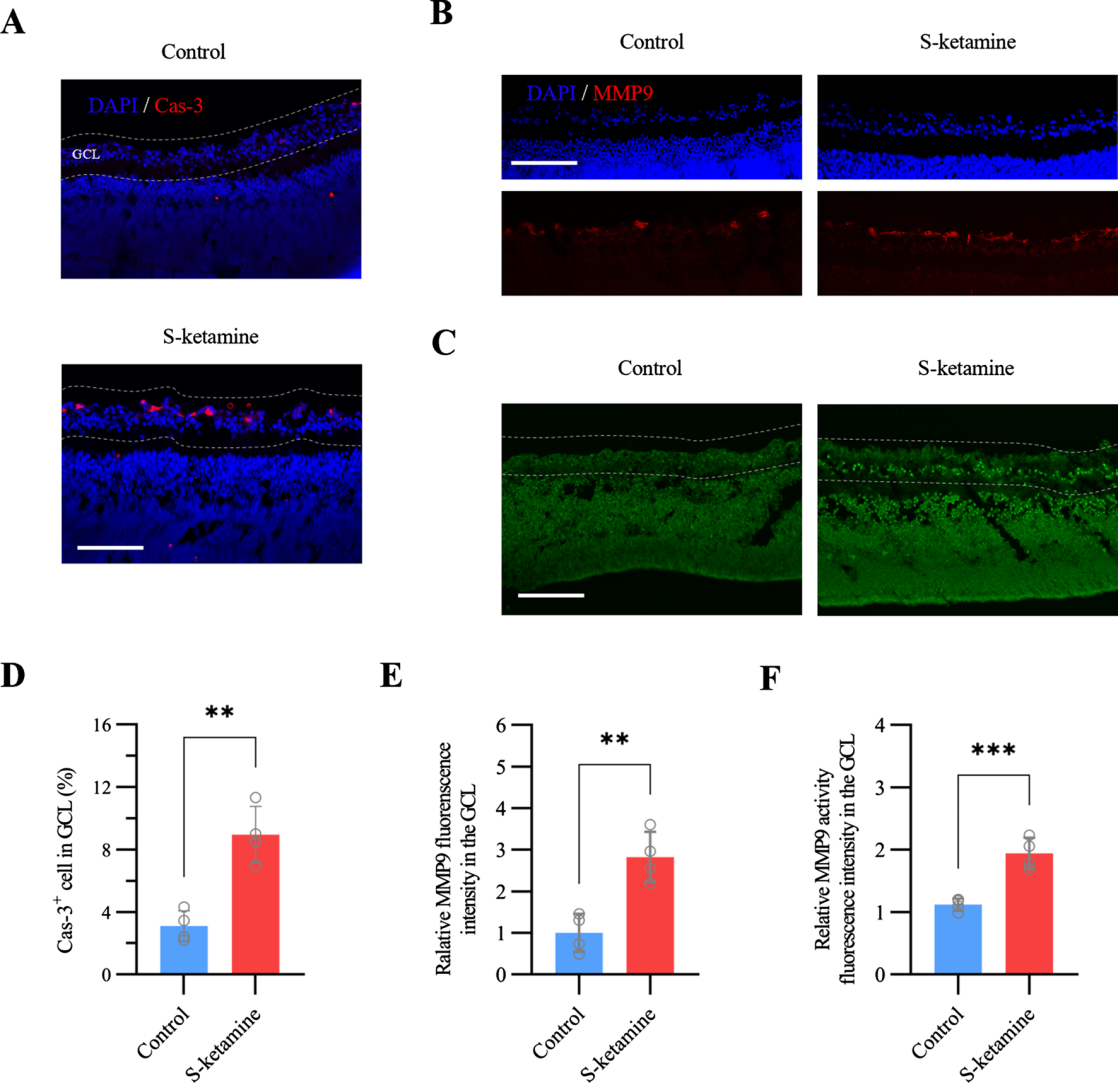
Increase of neuronal apoptosis and MMP9 expression/activity in retinal GCL after exposure to Esketamine for 5 h. **A**, **B** and **C** Representative images show active caspase 3 (Cas-3), MMP9 and MMP9 activity in situ zymography staining respectively in retinal GCL (area between two white dash lines) with and without esketamine exposure. Scale bar, 100 μm. (D) Bar graphs show the cells expressing cas-3 in GCL after esketamine exposure (n = 4 retinas per group). **E** and **F** The summary graph shows the fluorescence intensity of MMP9 expression (n = 4 retinas per group, E) and enzyme activity (n = 4 retinas per group, F) in GCL

Exposure to 150 μM esketamine for 5 h induced significant apoptosis in retinal GCL (Fig. 1A). The results showed that the percentage of Cas-3-immunostained apoptotic cells in the GCL increased from 3.1 ± 1.0% in control to 9.0 ± 1.9% in esketamine group (n = 4 retinas, two-tailed unpaired t test, *P* = 0.001, Fig. 1D). Concurrently, we found the MMP9 protein is expressed mainly in the GCL. The relative fluorescence intensity of MMP9 in GCL after esketamine exposure was approximately three-fold than in control group (n = 4 retinas, two-tailed unpaired t test, *P* = 0.003, Fig. 1B, E). Consistently, the intensity of enzyme activity by zymography in situ*.* in esketamine group shows two times higher than in control. (n = 4 retinas, two-tailed unpaired t test, *P* < 0.001, Fig. 1C, F). Taken together, these results suggest that esketamine exposure not only aggravates neuronal apoptosis, also upregulates the MMP9 expression and enzyme activity.

### Esketamine exposure increases colocalization of neurons and MMP9

**Fig. 2:**
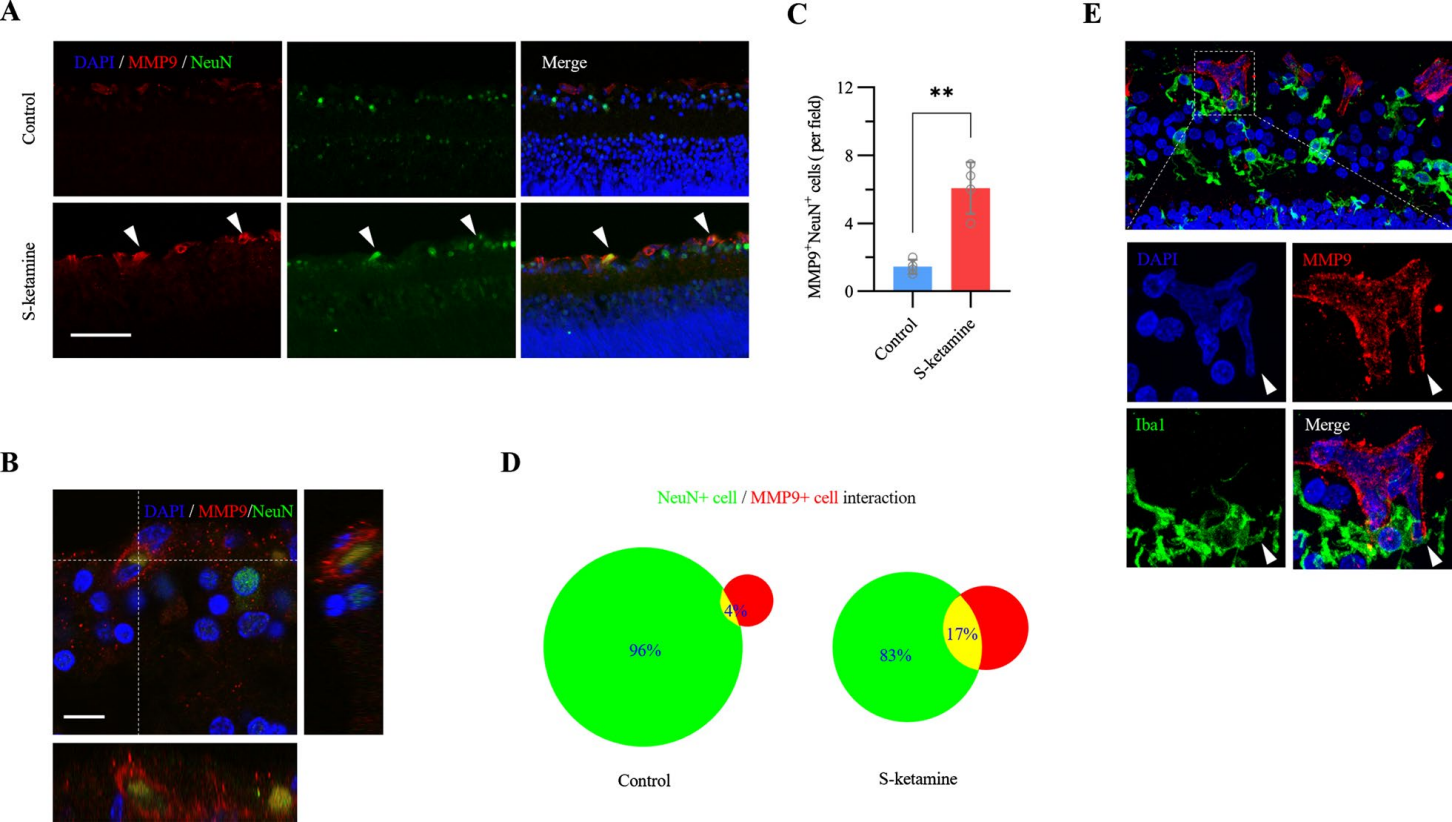
Increasing neurons in retinal GCL colocalized with MMP9 after esketamine exposure. **A** Representative images show immunostaining for MMP9 (red) and NeuN (green) in GCL with and without esketamine exposure. The arrowheads in white indicate ganglion cells (marked by NeuN) colocalized with MMP9. Scale bar, 50 μm. **B** The colocalization image is magnified. Scale bar, 20 μm. **C** Bar graph and **D** Venn diagram quantify the increase in overlap (neurons colocalized with MMP9 in GCL) from 4% in control to 17% after esketamine exposure (n = 4 retinas per group). **E** Representative images show immunostaining for MMP9 (red) and Iba1 (green) in GCL. Scale bar, 50 μm

To detect the spatial relationship between the MMP9 and retinal ganglion cells in GCL, we first colocalized MMP9 with NeuN^+^ cells (Fig. 2A, B, Additional file 1: Movie S1). Confocal images revealed the NeuN^+^ cells also stained by MMP9 were about 1.8 ± 0.8 cells / field in the control group. Esketamine exposure significantly increased the number of NeuN^+^MMP9^+^ cells to 6.1 ± 1.5 cells / field (n = 4 retinas, *P* < 0.001, two tailed unpaired t-test, Fig. 2C). The spatial overlap of NeuN^+^ and MMP9^+^ from 4% at baseline approached to 17% after esketamine exposure (Fig. 2D). Thus, these results indicated that the increasing apoptosis in GCL by esketamine may be a result of the increasing spatial interaction of MMP9 and cells. Previous study has reported microglia produced the early expression of MMP9 after injury [19]. Furthermore, our previous study found ketamine-induced microgliosis during the early retinal development [14]. To determine whether microglia is the potential source of MMP9, we additionally colocalized MMP9 and Iba1^+^ microglia (Fig. 2E). However, almost none of MMP9^+^ cells were co-labelled with Iba1^+^ microglia. Conversely, some of the MMP9^+^ cells in GCL were engulfed by microglia nearby. Taken together, these results suggest increasing MMP9 in GCL mediated esketamine-induced apoptosis, whereas microglia might play as a scavenger to clean the cells debris than producer of MMP9 after esketamine exposure.

### Esketamine causes Zn^2+^ time-dependent accumulation in GCL

**Fig. 3:**
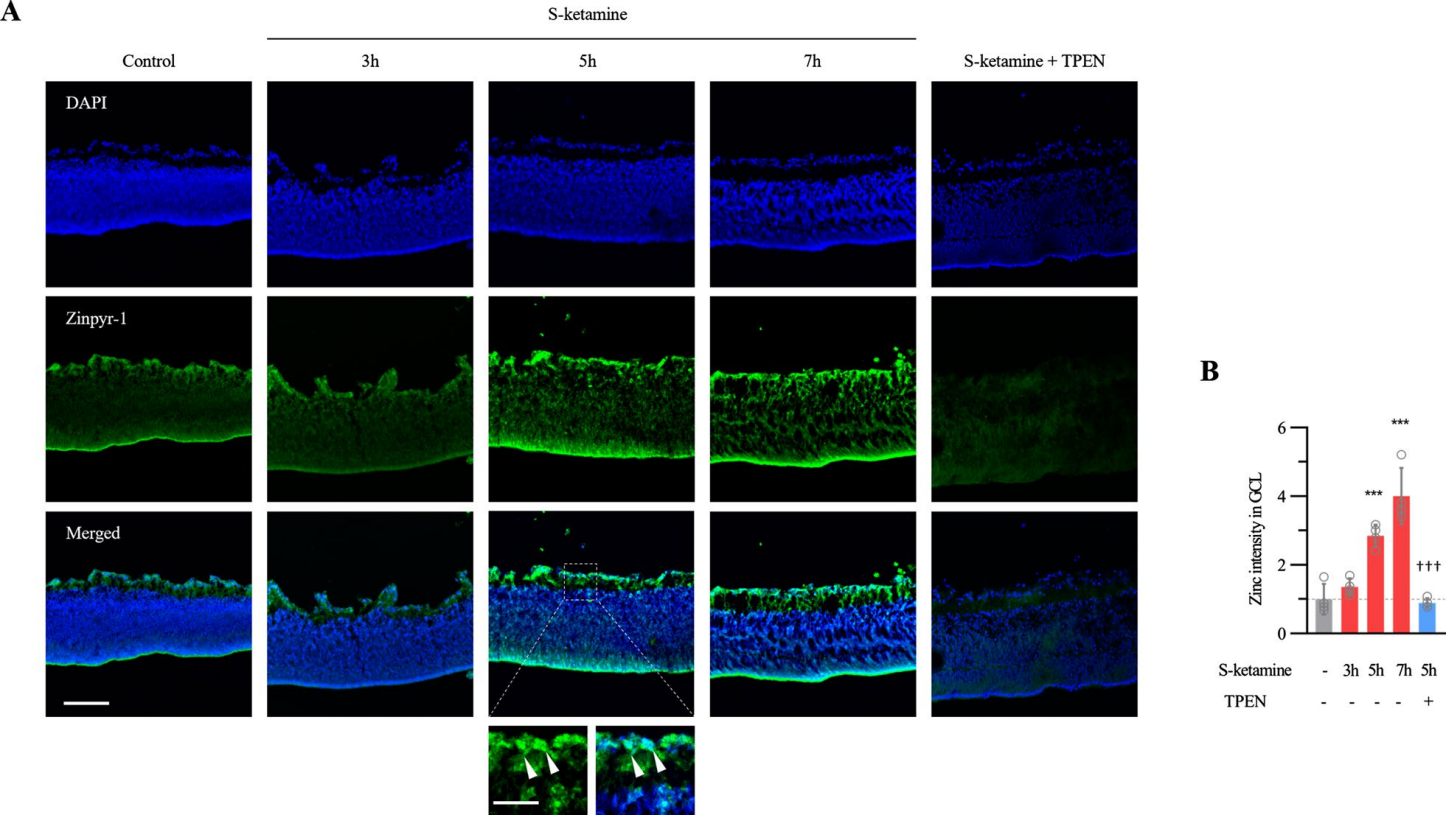
Time-dependent release of zinc after esketamine exposure. **A** Representative images show Zn^2+^ accumulation visualized in the whole-mount retina by the fluorescent Zn^2+^ sensor zinpyr-1. All images were taken at the same exposure. The image in the white dotted box is magnified as indicated with arrows. Scale bar, 100 μm. **B** The summary graph quantifies the intensity of zinpyr-1 signal in the GCL (n = 4 retinas per group). Grey bar: control; red bar: eyeballs exposure to esketamine for 3 h, 5 h and 7 h individually; blue bar: eyeballs exposure to TPEN and esketamine for 5 h. One-way ANOVA with Bonferroni post hoc tests. *** *P* < 0.001 compared with controls; ††† *P* < 0.001 compared with 5 h esketamine exposure

Zn^2+^ is necessary for enzyme activity of MMP9. To determine whether the Zn^2+^ was also raised after the esketamine exposure, we quantified the Zn^2+^ signal in situ at different timepoints after esketamine exposure. In normal retina, localization of Zn^2+^ by zinpyr-1 staining was primarily associated with GCL and to a similar extent with retinal pigment epithelial layer. Weaker Zn^2+^-zinpyr-1 fluorescence was found in other layers of the retina (Fig. 3A). Exposure to esketamine for 5 h increased the zinpyr-1 signal in GCL 2.9 ± 0.3-fold above baseline (n = 4 retinas per group, ANOVA with Bonferroni post hoc., adjusted *P* < 0.001, Fig. 3A, B). Zinpyr-1 signal in GCL demonstrated intracellular as well as extracellular matrix distribution. Considering that 5 h esketamine exposure significantly increased Zn^2+^ signal in GCL, we examined whether this signal could be eliminated by the high-affinity, cytomembrane-permeable Zn^2+^ chelator TPEN. Consequently, loss of zinpyr-1 signal in the whole-mount retina was observed when 100 μM TPEN was used (n = 4 retinas, 2.9 ± 0.3-fold *vs.* 0.9 ± 0.1-fold above baseline, ANOVA with Bonferroni post hoc., adjusted *P* < 0.001). Overall, these results suggest that esketamine exposure indeed causes Zn^2+^ accumulation in GCL.

### Albumin directly decreases zinc accumulation rather than through esketamine

**Fig. 4:**
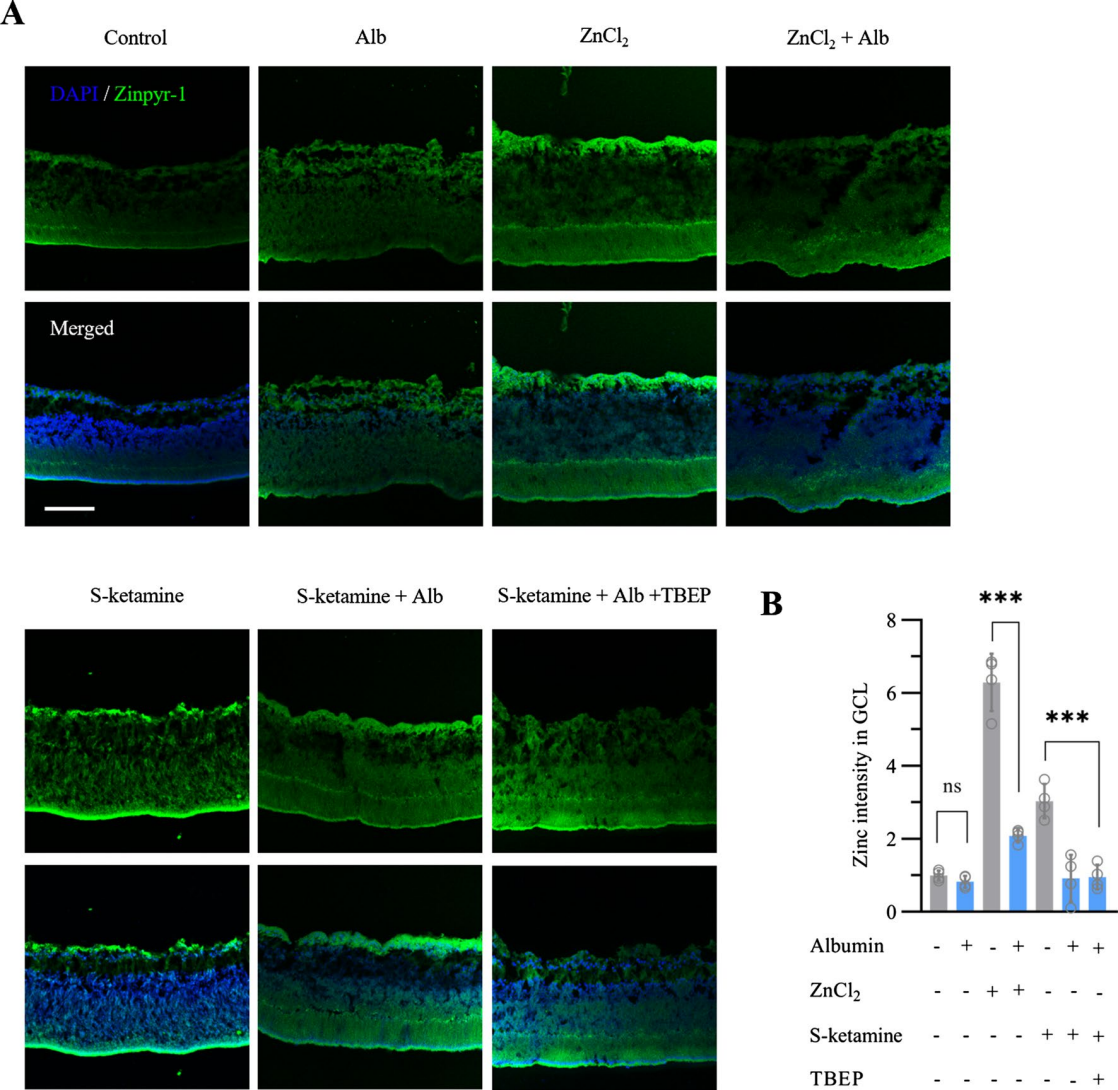
Effect of albumin on zinc after esketamine exposure. **A** Representative images show Zn^2+^ accumulation in retina by the fluorescent Zn^2+^ sensor zinpyr-1. All images were taken at the same exposure. Scale bar, 100 μm. **B** The summary graph quantifies the intensity of zinpyr-1 signal in the GCL (n = 4 retinas per group). *** *P* < 0.001 compared with non-albumin group

Albumin has potent binding capacity to Zn^2+^. We first investigated whether BSA can diminish the Zinpyr-1 signal produced by exogenous ZnCl_2_ (Fig. 4A, B). After incubation with 100 μM ZnCl_2_, the zinpyr-1 signal in retinal GCL raised to 6.3 ± 0.8-fold above baseline (n = 4 retinas, ANOVA with Bonferroni post hoc., adjusted *P* < 0.001). Concurrently, treatment with 5% albumin strongly suppressed but not fully eliminated the zinpyr-1 signal (n = 4 retinas, 2.1 ± 0.2-fold *vs.* 6.3 ± 0.8-fold above baseline, ANOVA with Bonferroni post hoc., adjusted *P* < 0.001). We further examined whether BSA had similar effect on elimination of zinpyr-1 signal after esketamine exposure. In fact, besides interacting with Zn^2+^, BSA also has esketamine-albumin interaction at different site [20]. Therefore, TBEP was used to abolish the binding of esketamine to albumin [21]. After incubation with 10 μg/ml TBEP and albumin, the zinpyr-1 signal raised by esketamine faded obviously and approached to baseline (n = 4 retinas, 1.0 ± 0.3-fold in esketamine + TBEP + albumin (KTA) group *vs.* 3.0 ± 0.5-fold above baseline in esketamine group, ANOVA with Bonferroni post hoc., adjusted *P* < 0.001). Taken together, these results indicated albumin can effectively chelate endogenous Zn^2+^ after esketamine exposure.

### Albumin downregulates MMP9 and decreases M1 microglia

**Fig. 5:**
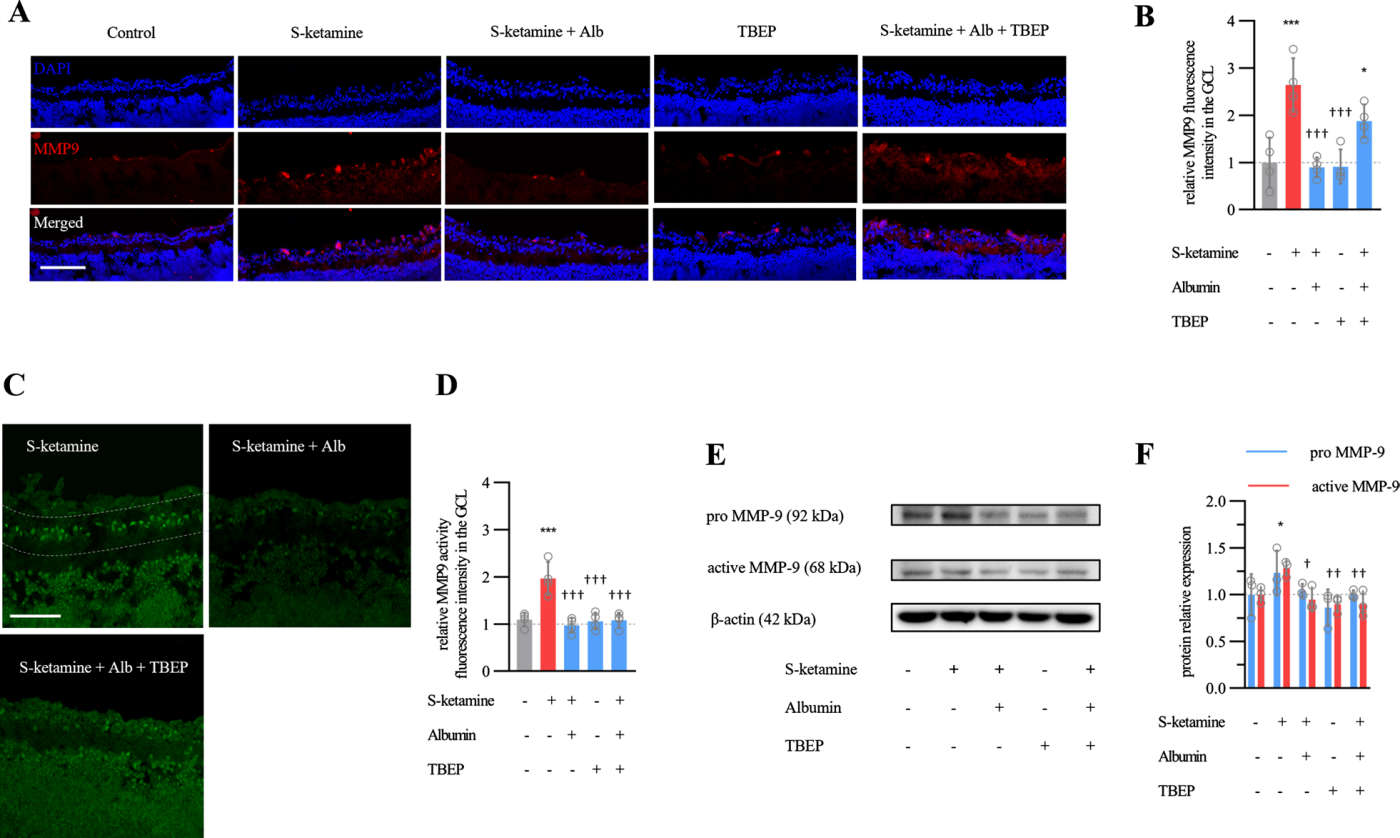
Effect of albumin on MMP9 expression and enzyme activity in GCL. **A** Representative images show MMP9 histoimmunostaining in retinal. Scale bar, 100 μm. **B** Bar graph shows the shows the fluorescence intensity of MMP9 expression (n = 4 retinas per group). **C** Representative images show MMP9 activity in situ zymography staining in GCL. Scale bar, 50 μm. **D** Bar graph shows the fluorescence intensity of MMP9 activity in GCL (n = 4 retinas per group). **E** and **F** Representative images of western blot and bar graph summary show pro MMP9 (92 kDa) and active MMP9 protein (68 kDa) expression in whole retina tissue (n = 3 retina per group). The full-length blots are presented in Additional file 2: Figure S1. * *P* < 0.05, *** *P* < 0.001 compared with control group; † *P* < 0.05, ††† *P* < 0.001 compared with esketamine group

We further determined whether albumin also inhibited the MMP9 expression and enzyme activity. After semi-quantification by immunohistostaining, we found the intensity of MMP9 in GCL was decreased from 2.6 ± 0.6-fold above baseline in esketamine group to 1.88 ± 0.4-fold in KTA group (n = 4 retinas, ANOVA with Bonferroni post hoc., adjusted *P* < 0.001, Fig. 5A, B). Additionally, we testified MMP9 enzyme activity after in situ gelatin substrate zymography. As a result, the MMP9 activity in GCL was decreased from 1.97 ± 0.3-fold above baseline in esketamine group to 1.08 ± 0.1-fold in KTA group (n = 4 retinas, ANOVA with Bonferroni post hoc., adjusted *P* < 0.001, Fig. 5C-D). To confirm the change of MMP9 protein and enzyme activity, additional western blotting was employed to measure the pro (zymogen, 92 kDa) and active (cleaved, 68 kDa) MMP9 protein in the whole retina. Consistent with the results by immunohistochemistry and in situ gelatin substrate zymography, both pro and active MMP9 were reduced significantly in KTA group than in esketamine group (n = 3 retinas, ANOVA with Bonferroni post hoc., adjusted *P* < 0.01, Fig. 5E, F). Taken together, these results support the conclusion that 5% BSA is not only able to decrease the MMP9 expression, but also inhibit enzyme activity via chelation of Zn^2+^.

**Fig. 6:**
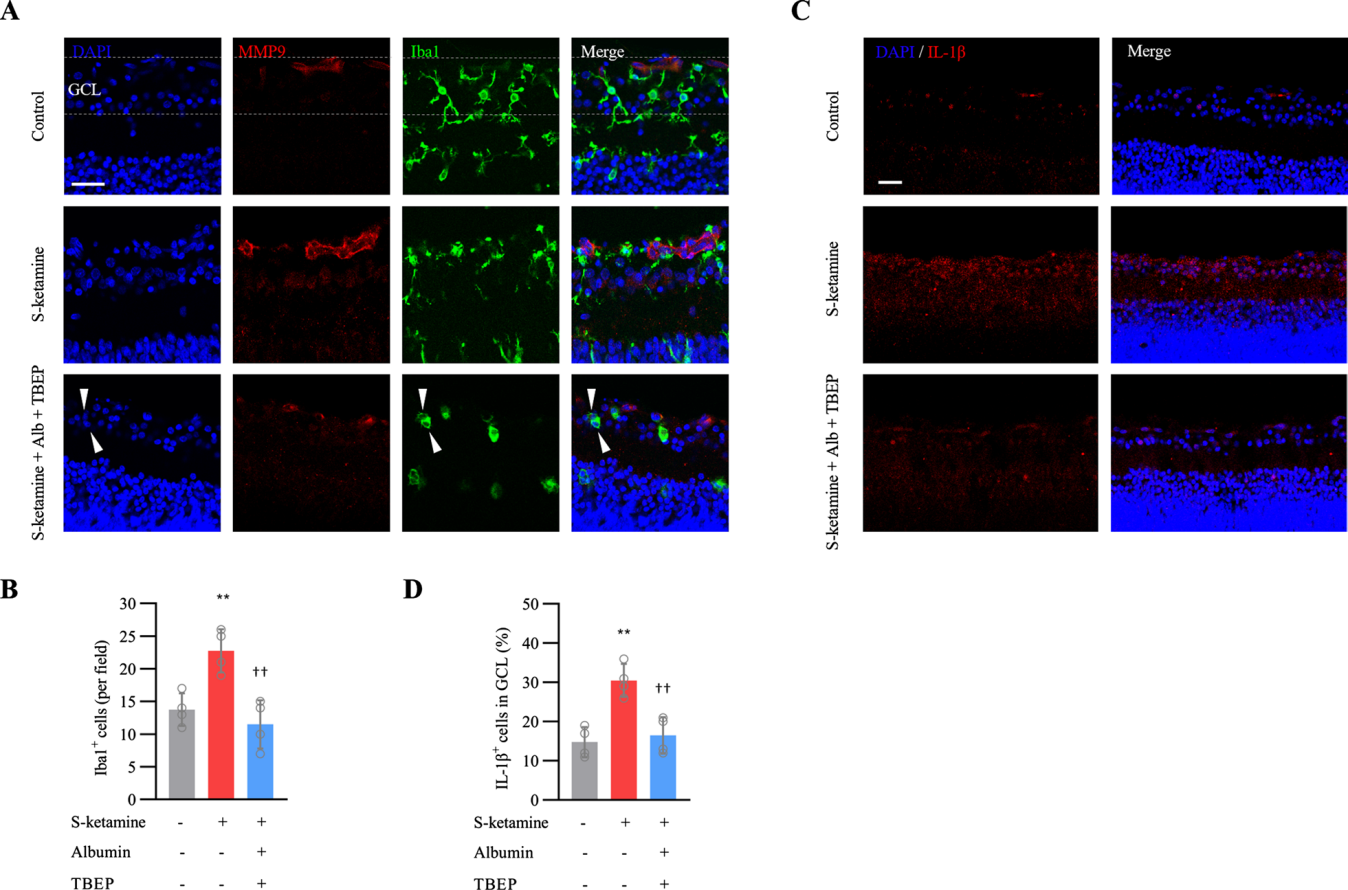
Effect of albumin on microglia and IL-1β in GCL. **A** Representative images show Iba1 staining respectively in retinal GCL. Scale bar, 10 μm. **B** Bar graphs show the cells expressing Iba1 in GCL (n = 4 retinas per group). **C** Representative images show IL-1β staining respectively in retinal GCL. Scale bar, 20 μm. **D** Bar graphs show the cells expressing IL-1β in GCL (n = 4 retinas per group). ** *P* < 0.01 compared with controls; †† *P* < 0.01 compared with esketamine

Ketamine induced neuronal apoptosis and promoted pro-inflammatory microglia (M1) phenotypes, despite the later seems independent of increasing MMP9. In the CNS, albumin may attenuate microglial activation and modulate the inflammatory cytokines [22, 23]. To explore the effect of BSA on microgliosis secondary to ketamine exposure, we analyzed the microglia in density and morphology. As shown in Fig. 6A, exposure to esketamine for 5 h significantly increased microglia from 14 ± 3 cells / field in control group to 22 ± 3 cells / field (n = 4 retinas, ANOVA with Bonferroni post hoc., adjusted *P* = 0.008, Fig. 6B). Meanwhile, most of microglia switched from a “resting” ramified morphology in normal P7 retina to an “active” amoeboid-like macrophage, which is characterized by a large and rounded soma with shorten or ablated process. Compared to esketamine, treatment with 5% BSA decreased microglia to 12 ± 4 cells / field (n = 4 retinas, ANOVA with Bonferroni post hoc., adjusted *P* = 0.002). Most importantly, almost all Iba1^+^ cells displayed multi-nucleated somas without any process (Fig. 6A), suggesting BSA might promote microglia transformation to the macrophagocyte or macrophagocyte fusion with each other. To confirm whether pro-inflammatory effect of “M1” microglia disappeared along with morphological change, we quantified the “M1” subtype marker IL-1β (Fig. 6C). As a result, BSA treatment also decreased IL-1β expression, which was up-regulated strikingly after esketamine exposure (Fig. 6D). Taken together, transformation from pro-inflammatory microglia to multi-nuclear macrophagocytes after BSA treatment may be a way to clean up the area without the debris and without inflammation.

### Albumin alleviates the apoptosis in retinal GCL

**Fig. 7:**
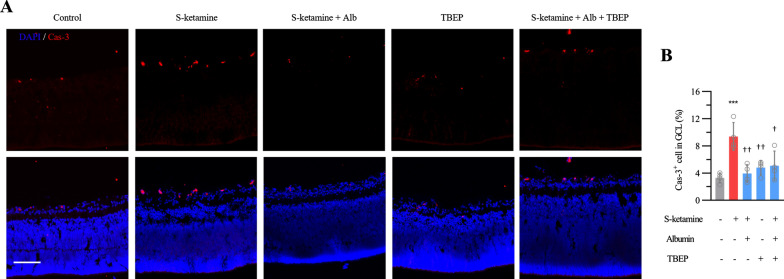
Protective effect albumin on cells in retinal GCL against apoptosis after esketamine exposure. **A** Representative images show active caspase 3 (Cas-3) staining respectively in retinal GCL. Scale bar, 100 μm. **B** Bar graphs show the cells expressing cas-3 in GCL (n = 4 retinas per group). *** *P* < 0.001 compared with control group; † *P* < 0.05, †† *P* < 0.01 compared with esketamine group

We confirmed albumin can ameliorate Zn^2+^-dependent upregulation of MMP9 after esketamine exposure. Therefore, we further testified the protective effect of albumin on esketamine-induced apoptosis (Fig. 7A). The percentage of Cas-3-immunostained apoptotic cells in the GCL decreased from 9.4 ± 2.1% in the esketamine to 5.1 ± 2.2% in KTA group (n = 4 retinas, ANOVA with Bonferroni post hoc*.*, adjusted *P* = 0.01, Fig. 7B).

## Discussion

In the present study, we demonstrated that esketamine exposure caused Zn^2+^ accumulation and Zn^2+^-dependent upregulation of MMP9 protein expression and enzyme activity in the GCL of P7 rat retina, which eventually aggravated neuronal apoptosis of GCL during the early development. Esketamine exposure simultaneously caused microglia activation and MMP9 upregulation in rat retina, but both of them were not related. Albumin, a therapeutic agent commonly used for fluid resuscitation during anesthesia, ameliorated esketamine-induced neuronal apoptosis by decreasing the accumulation of Zn^2+^ in a time-dependent manner and downregulating the MMP9 protein expression and enzyme activity. Furthermore, the decrease of Zn^2+^ accumulation might not be due to the reduction of free esketamine since blockade the binding of esketamine to albumin also reduced Zn^2+^ accumulation to the similar degree.

Similar to racemic ketamine, esketamine as the S-enantiomer of traditional general anesthetic ketamine not only produces fast and effective general anesthesia, but also causes neuronal apoptosis during the early development. Furthermore, our study showed that esketamine induced about 50% increase of neuronal apoptosis than racemic ketamine at P7 rat retina, which might be due to the higher affinity to NMDA receptor than ketamine [24]. Preservative benzethonium in racemic ketamine plays an additive role in neurotoxicity [25], our study indicated that preservative-free esketamine played a predominant role triggering neuronal apoptosis during neuronal development.

Apart from neuronal apoptosis, our study found that esketamine exposure also caused microglia activation and MMP9 upregulation simultaneously. Many studies including our own studies have repetitively demonstrated that inflammatory cytokines release from activated microglia and ECM degradation by upregulated MMP9 expression are involved in neuronal apoptosis. Previous studies have demonstrated that activated microglia is the source of MMP9 and concentration of MMP9 protein is upregulated in the supernatants from LPS-stimulated microglia [26], indicating that upregulated MMP9 expression is released by activated microglia. However, MMP9 in current study had closer spatial relationship with neuron than microglia. In addition to activated microglia, upregulated MMP9 expression may be also derived from neurons, astroglia, etc. cell types [27]. The sources of MMP9 expressions after esketamine exposure need to be further investigated.

It is well known that the activity of MMP9 is dependent on free Zn^2+^. Our study showed that Zn^2+^ signal in P7 rat retina was significantly increased along with MMP9 activity and protein expression upregulation and RGCs apoptosis after esketamine exposure for 5 h. Furthermore, Zn^2+^ signal was mainly distributed in GCL and retinal pigment epithelial layer. We did not further study the source of Zn^2+^, and Li et al. found that Zn^2+^ quickly increases in the inner- and outermost sublaminae of the retinal inner plexiform layer after mouse optic nerve crush and then transfers via vesicular release to RGCs [28]. Furthermore, intraocular Zn^2+^ chelation attenuates RGC death. Therefore, it is possible that Zn^2+^ in RGCs might come from retinal pigment epithelial layer and inner plexiform layer and involved in esketamine-induced neuronal apoptosis in P7 rat retina through upregulating MMP9 activity. Whereas in adult retina the Zn^2+^ signal appears first in IPL and then GCL after injury [29, 30]. It might be related with the differentiation of INL and maturation of synaptic connect along with the development [31].

Mounting studies show that TPEN as a Zn^2+^ chelator can attenuate injury or ketamine-induced neuronal apoptosis, but TPEN cannot be used in clinic [15]. Albumin is not only an effective therapeutic agent for fluid resuscitation, but it is also an effective binding agent for free Zn^2+^ and esketamine. Our study found albumin decreased the Zn^2+^ accumulation in GCL and ameliorated esketamine-induced neuronal apoptosis. To rule out the possibility that the amelioration of neuronal apoptosis is not caused by the reduction of free concentration of esketamine due to the binding between esketamine and albumin, we used TBEP to block the binding of esketamine to albumin. The results found that the apoptosis in GCL was still ameliorated significantly, indicating that the protective effect of albumin in esketamine induced-neuronal apoptosis is the result of decrease in free Zn^2+^ but not free esketamine.

In addition to the ability of chelating free Zn^2+^, albumin also demonstrates anti-inflammation properties, like in degenerative diseases [32, 33]. In our study, we found that microglia were decreased and switched into multi-nucleated macrophagocyte after treated with albumin. Microglia might be transformed to multi-nucleated macrophagocytes cleaning the cell debris and inflammation, finally return to ramified “resting” morphology [34].

Most of animal studies have shown albumin to be a promising neuroprotectant in cerebral ischemia, traumatic brain injury and neurodegenerative disease [23, 32, 35]. The binding of esketamine to albumin reduced the free esketamine, which alleviated the neurotoxicity. After blockade of the interaction between esketamine and albumin, apoptosis in GCL still declined. It indicated protective effect of albumin is the result of decrease in free Zn^2+^. Meanwhile, albumin demonstrated anti-inflammation properties, like in degenerative diseases [32, 33].

Despite we found neuroprotection of albumin, current results should be explained with caution because therapeutic effect of albumin was testified in animal study. Clinical trials for ischemic stroke demonstrated high dose albumin therapy but have no benefit, and it even increased myocardial stress [36]. On the other hand, it is still unclear whether blood–brain barrier is injured after esketamine exposure and brain parenchyma accessible for albumin. Thus, the long-term outcome of albumin in vivo requires further elucidation, especially in pediatric patients who undergo repeated general anesthesia.

In conclusion, albumin can chelate Zn^2+^ and down-regulate MMP9, ultimately attenuate neuronal apoptosis in the retinal GCL during the early development.

## Supplementary Information

**Additional file 1: Movie S1.** Neurons marked by NeuN (green) in retinal GCL colocalized with MMP9 (red) after esketamine exposure.

**Additional file 2: Figure S1.** The full-length and unprocessed blots showed pro MMP9 (92 kDa) and active MMP9 protein (68 kDa) expression in whole retina tissue.

## Data Availability

The datasets used and/or analyzed during the current study are available from the corresponding author on reasonable request.

## Abbreviations

ACSF: Artificial cerebrospinal fluid
ANOVA: Analysis of variance
BSA: Bovine serum albumin
ECM: Extra-cellular environment
GABA_A_: γ-Aminobutyric acid type A
HSA: Human serum albumin
IL-1β: Interleukin 1β
MMP9: Matrix metalloproteinase 9
NMDA: N-Methyl-D-aspartate
TBEP: Tris (2-butoxyethyl) phosphate
