# Supplementary material for: Albumin alleviated esketamine-induced neuronal apoptosis of rat retina through downregulation of Zn2+-dependent matrix metalloproteinase 9 during the early development
Source: BMC Neurosci. 2022 Nov 16;23:66. doi: 10.1186/s12868-022-00753-5 (PMC9670403; doi:10.1186/s12868-022-00753-5)
Supplement: Supplementary file 2 — Additional file 2: Figure S1. The full-length and unprocessed blots showed pro MMP9 (92 kDa) and active MMP9 protein (68 kDa) expression in whole retina tissue. [file 12868_2022_753_MOESM2_ESM.docx]

Supplementary File

Albumin alleviated esketamine-induced neuronal apoptosis of rat retina through downregulation of Zn^2+^-dependent matrix metalloproteinase 9 during the early development

Kan Zhang ^1,2^, Ruijing Ma ^3^, Luping Feng ^2^, Peiwen Liu ^1,2^, Shuang Cai ^1,2^, Chaoyang Tong ^1,2^, Jijian Zheng ^1,2^

Corresponding author:

Jijian Zheng, (Email: zhengjijian626@sina.com)

This supplementary file includes Movie 1and Figure S1.

Movie 1. Neurons marked by NeuN (green) in retinal GCL colocalized with MMP9 (red) after esketamine exposure.


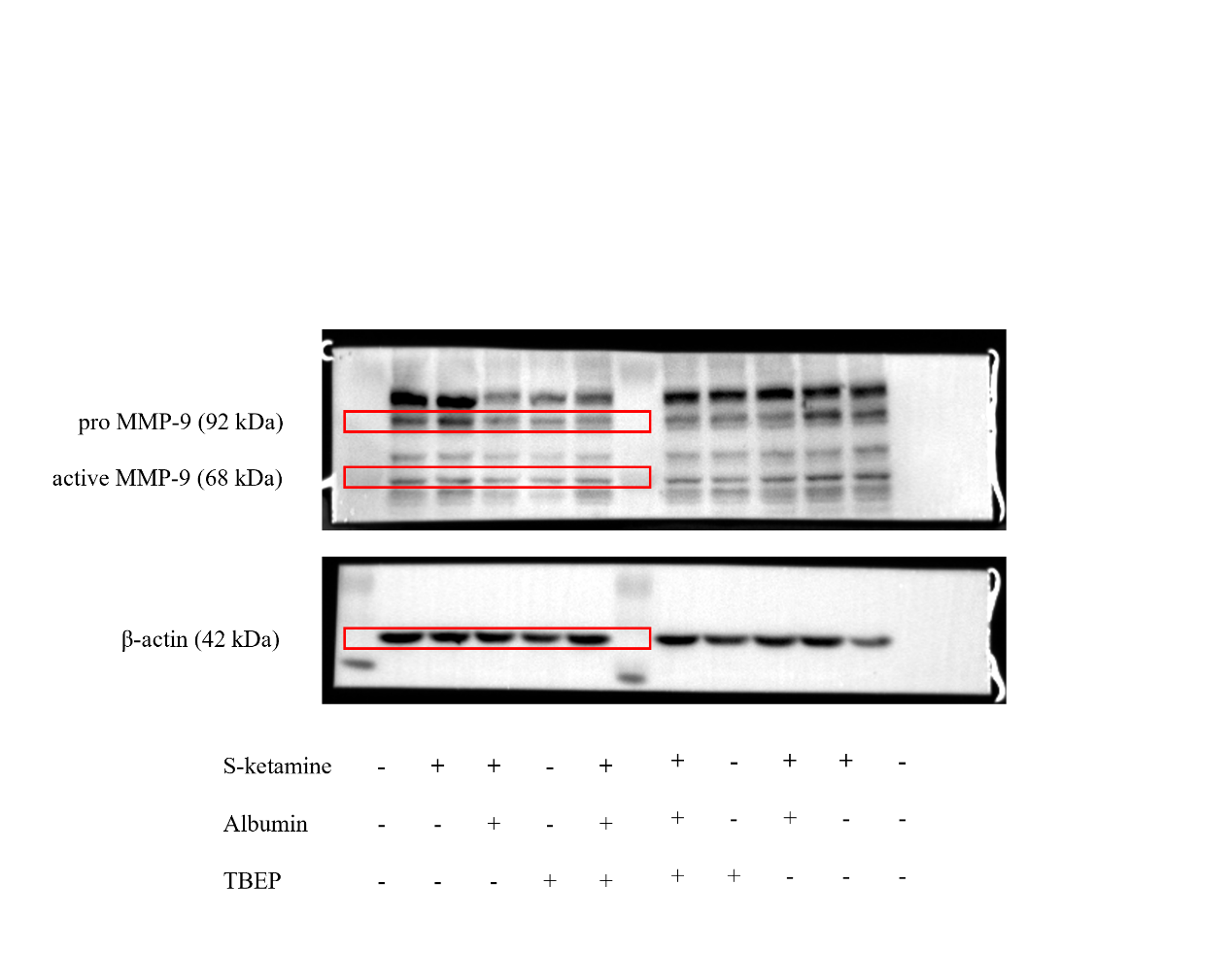


Figure S1. The full-length and unprocessed blots showed pro MMP9 (92 kDa) and active MMP9 protein (68 kDa) expression in whole retina tissue.
